# Supplementary material for: Spatial Transcriptomics-correlated Electron Microscopy maps transcriptional and ultrastructural responses to brain injury
Source: Nat Commun. 2023 Jul 11;14:4115. doi: 10.1038/s41467-023-39447-9 (PMC10336148; doi:10.1038/s41467-023-39447-9)
Supplement: Supplementary file 1 — Supplementary Information [file 41467_2023_39447_MOESM1_ESM.pdf]

Supplementary Information

Spatial Transcriptomics-Correlated Electron Microscopy maps transcriptional and ultrastructural responses to brain injury

Peter Androvic[1§], Martina Schifferer,[2,3§], Katrin Perez Anderson[1], Ludovico Cantuti-Castelvetri[2,4], Hanyi Jiang[2,3], Hao Ji[1], Lu Liu[1], Garyfallia Gouna[2,4], Stefan A. Berghoff[2,4], Simon Besson-Girard[1], Johanna Knoferle[2,4], Mikael Simons[1,2,3,4], Ozgun Gokce[1,3,5] *

[1]Institute for Stroke and Dementia Research, University Hospital of Munich, LMU Munich, Munich, Germany
[2]German Center for Neurodegenerative Diseases (DZNE), Munich, Germany
[3]Munich Cluster of Systems Neurology (SyNergy), Munich, Germany
[4]Institute of Neuronal Cell Biology, Technical University Munich, Munich, Germany
[5]Department of Neurodegenerative Diseases and Geriatric Psychiatry, University Hospital Bonn, Bonn, Germany
[§]Equal contribution

*Corresponding: O. Gokce (Ozgun.Goekce@ukbonn.de)

CONTENT:

Supplementary figures S1 – S9

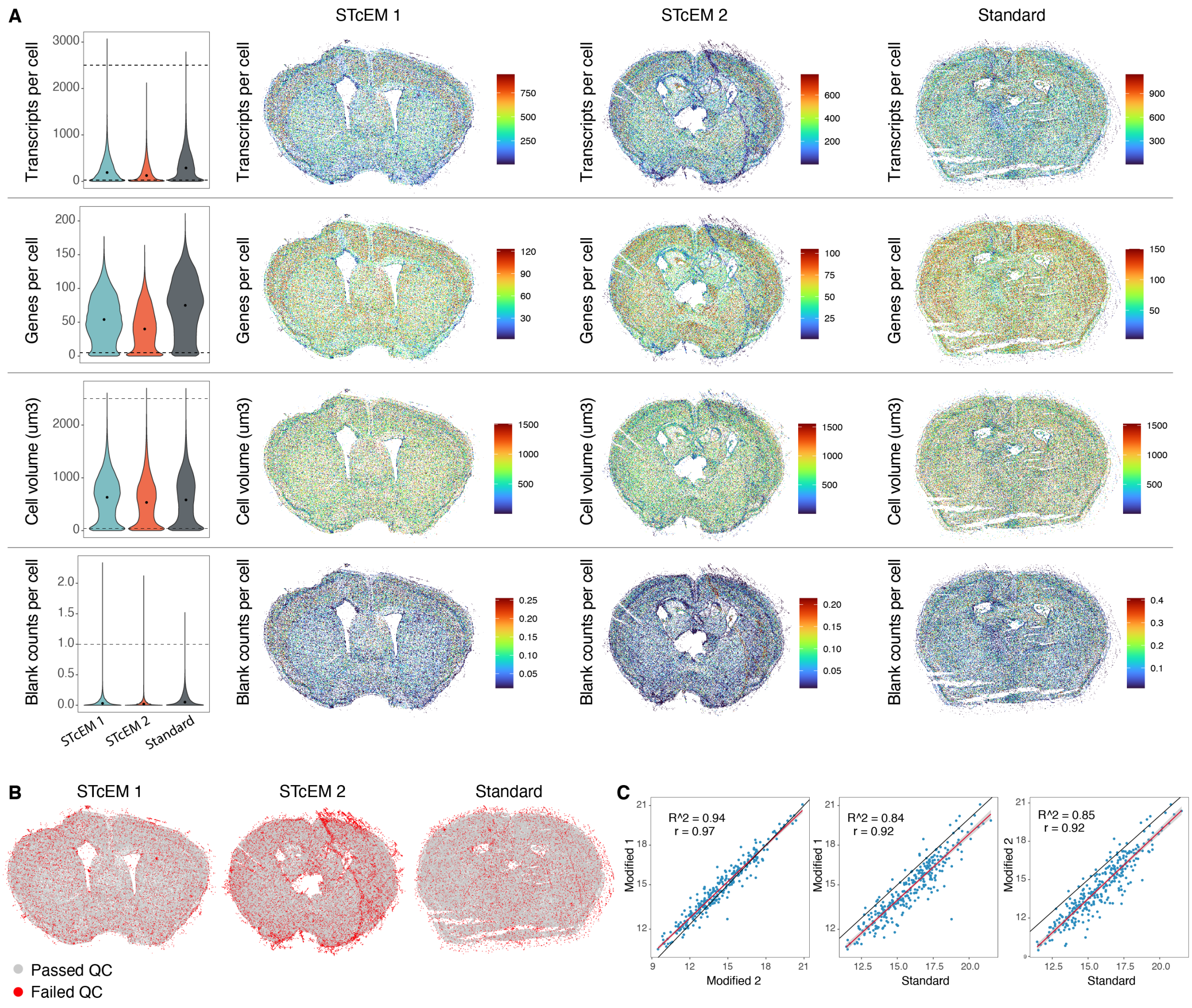

Figure S1. MERFISH quality control (QC). **A** QC metrics of MERFISH data in measured brain sections. Dotted lines in violin plots represent cutoff thresholds. Point shows median value. Brain sections on the right show distribution of QC metric values in space. **B** Spatial plots showing location of cells passing or failing quality control. **C** Correlation of gene expression values between each measured section.

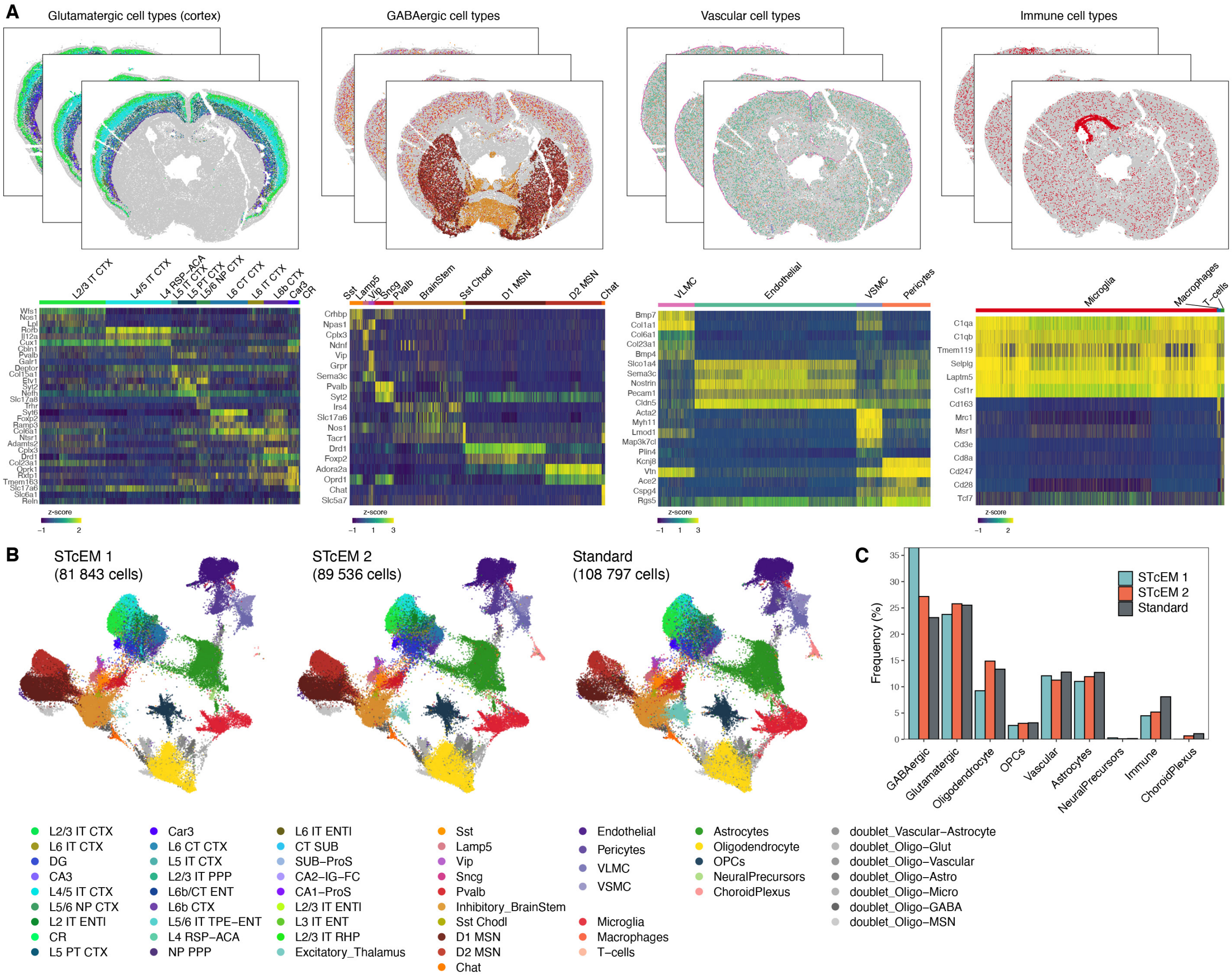

Figure S2. Extended cell type annotation. A Spatial plots showing location of cell types belonging to major cell classes (top) and expression of their markers (bottom heatmaps). **B** UMAP plots split by section and colored by identified cell types. **C** Barplot showing frequency of major cell classes per measured section.

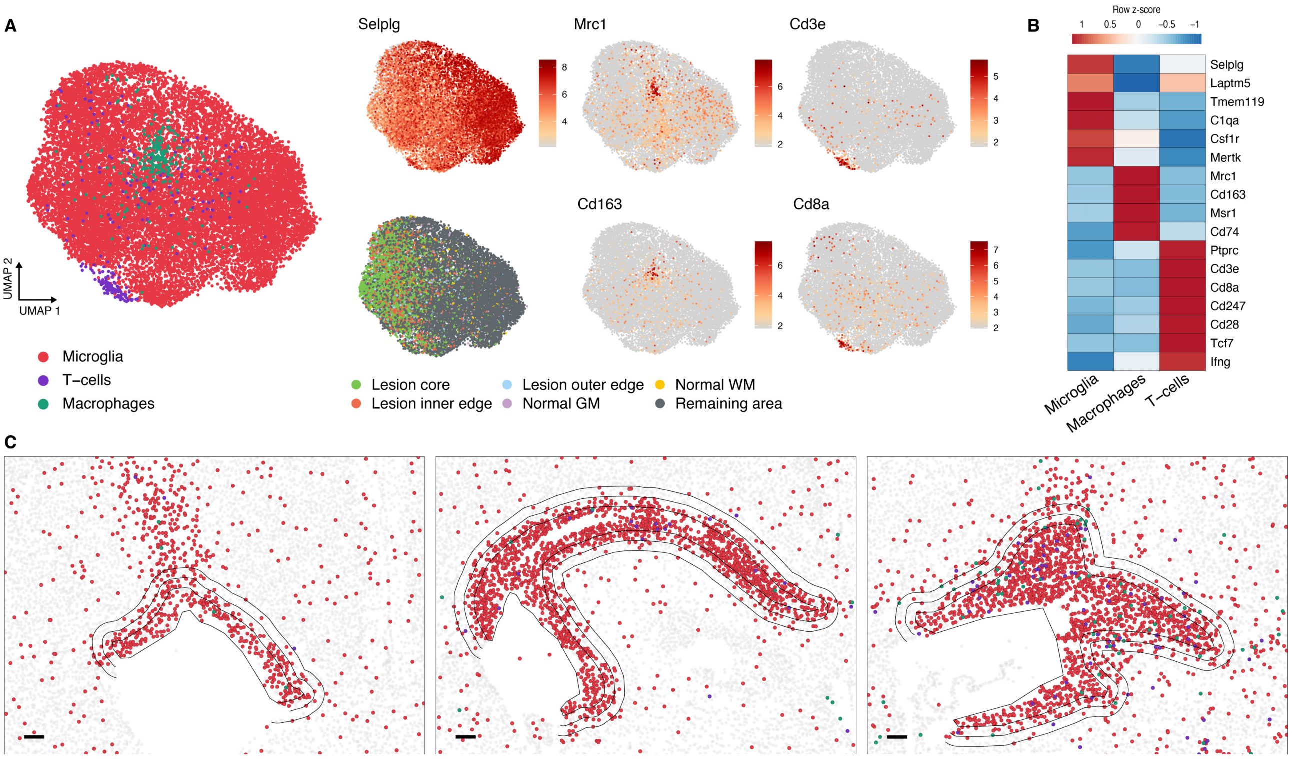

Figure S3. MERFISH analysis of immune cells. A UMAP plot of immune cells identified by MERFISH colored by cell type (left), experimental group, and expression of selected markers (right). **B** Heatmap of average expression per immune cell type. **C** MERFISH spatial plots of immune cell types in the lesion area of three biological replicate sections. Polygons depict segmented lesion areas. Scale bar 100 µm.

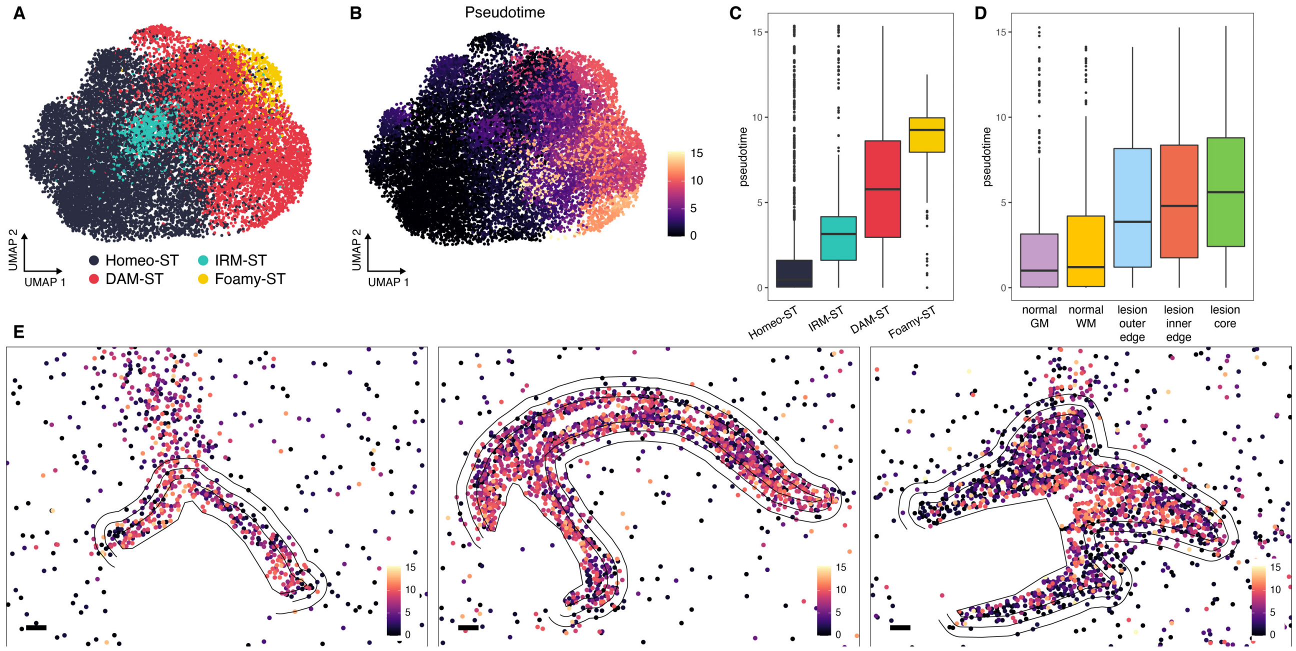

Figure S4. Pseudotime analysis. A UMAP plots of microglia colored by identified clusters (left), tissue region, and expression of marker genes (right). DAM = disease-associated microglia, IRM = interferon-responsive microglia. **B** UMAP plots of microglia colored by pseudotime value. **C** Boxplot of pseudotime values across microglia clusters. N=1017 cells obtained from 8 animals. **D** Boxplot of pseudotime values across segmented tissue regions. N=1017 cells obtained from 8 animals. Boxplots display the median (central line), interquartile range (IQR; box), and 1.5 * IQR (whiskers). Points beyond the whiskers represent outliers. **E** Spatial plot of pseudotime values in microglia in the lesion area.

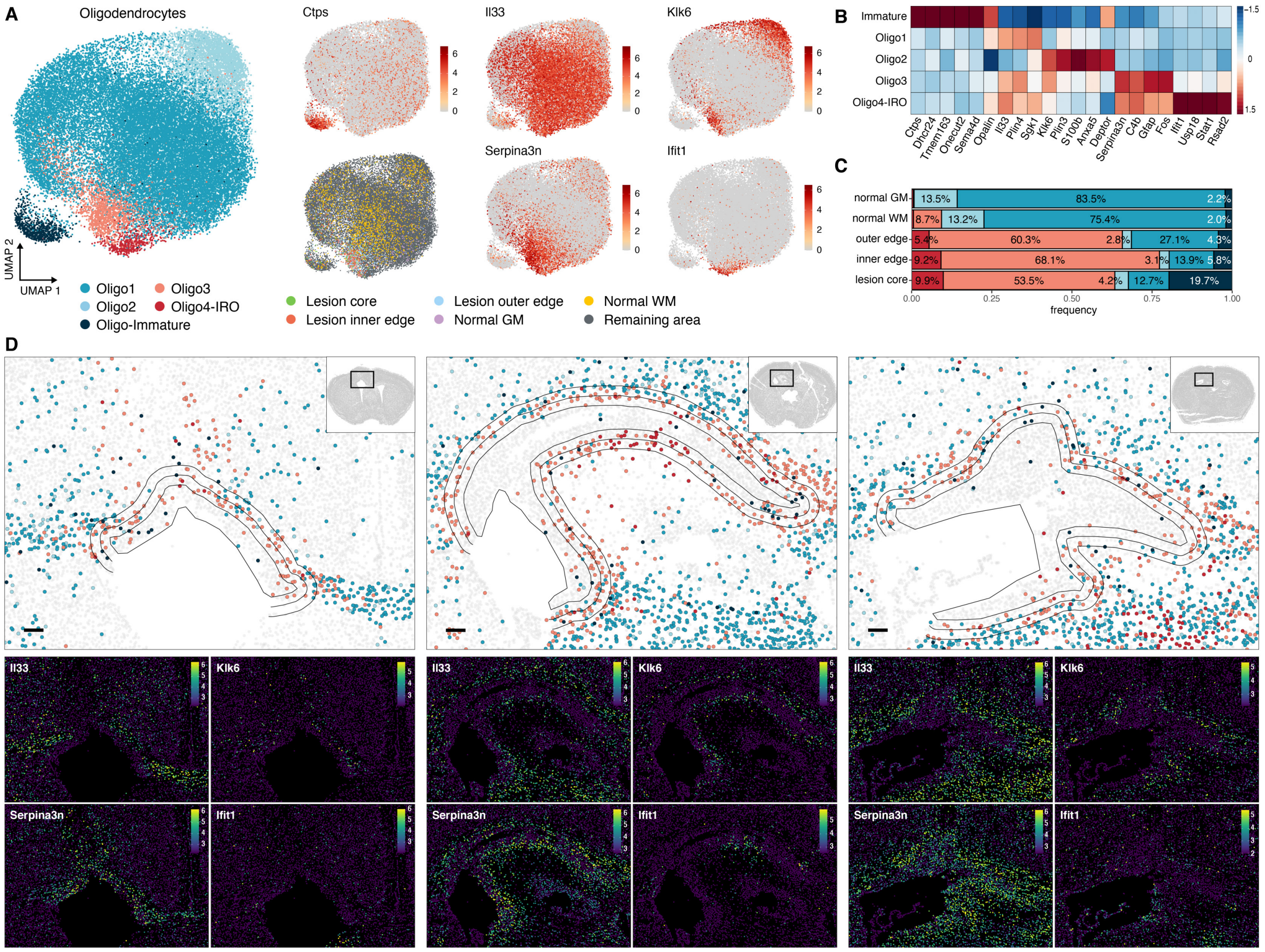

Figure S5. MERFISH analysis of oligodendrocytes. A UMAP plots of oligodendrocytes colored by identified clusters (left), tissue region, and expression of marker genes (right). IRO = Interferon-responsive oligodendrocytes. **B** Heatmap of average expression per oligodendrocyte cluster. **C** Frequency of oligodendrocyte clusters per tissue region. **D** MERFISH spatial plots of oligodendrocyte clusters in the lesion area of three biological replicate sections (top) and spatial expression of selected marker genes (bottom). Polygons depict segmented lesion areas. Scale bar 100 µm.

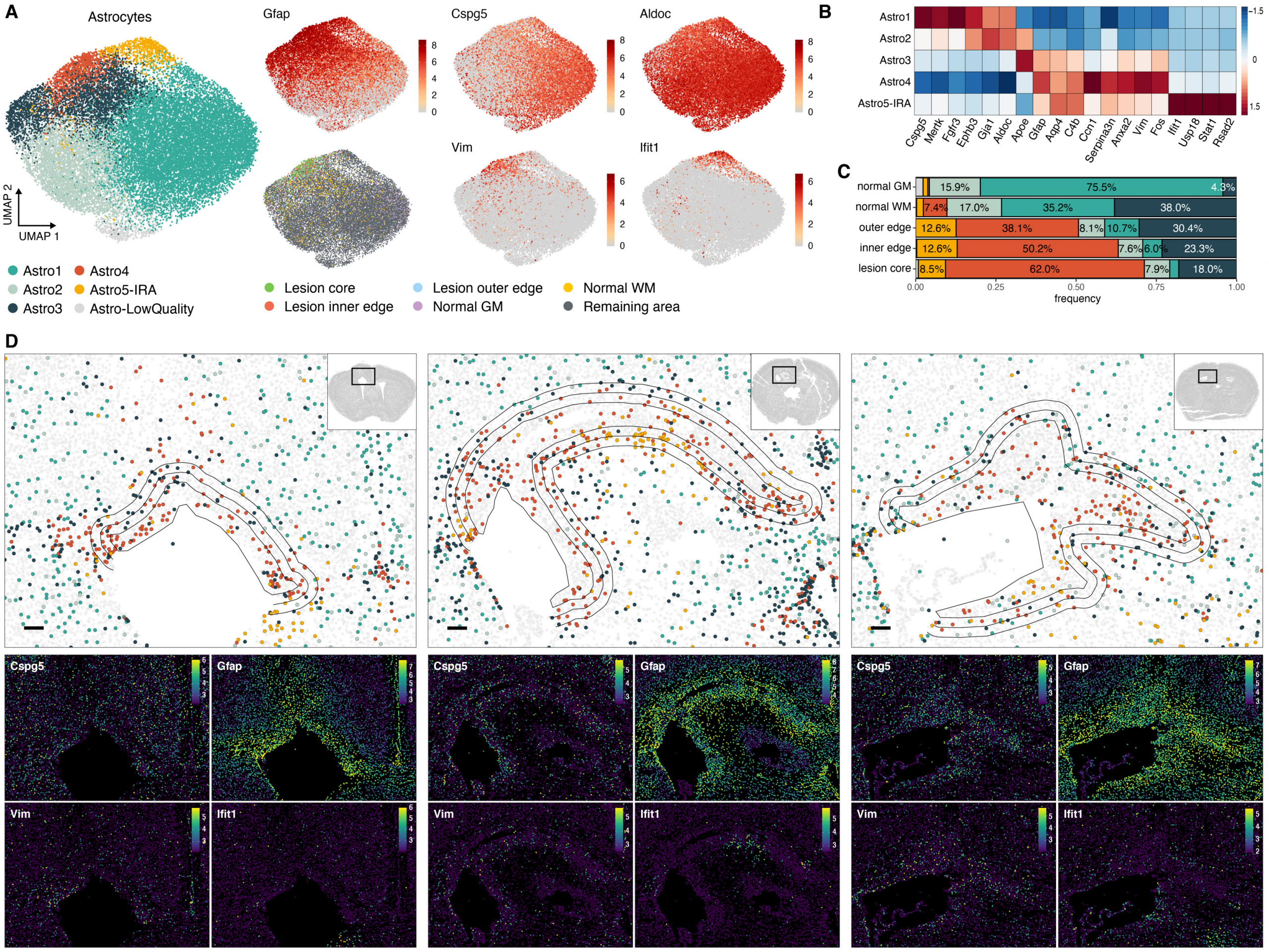

Figure S6. MERFISH analysis of astrocytes. A UMAP plots of astrocytes colored by identified clusters (left), tissue region, and expression of marker genes (right). IRA = Interferon-responsive astrocytes. **B** Heatmap of average expression per astrocyte cluster. **C** Frequency of astrocyte clusters per tissue region. **D** MERFISH spatial plots of astrocyte clusters in the lesion area of three biological replicate sections (top) and spatial expression of selected marker genes (bottom). Polygons depict segmented lesion areas. Scale bar 100 µm.

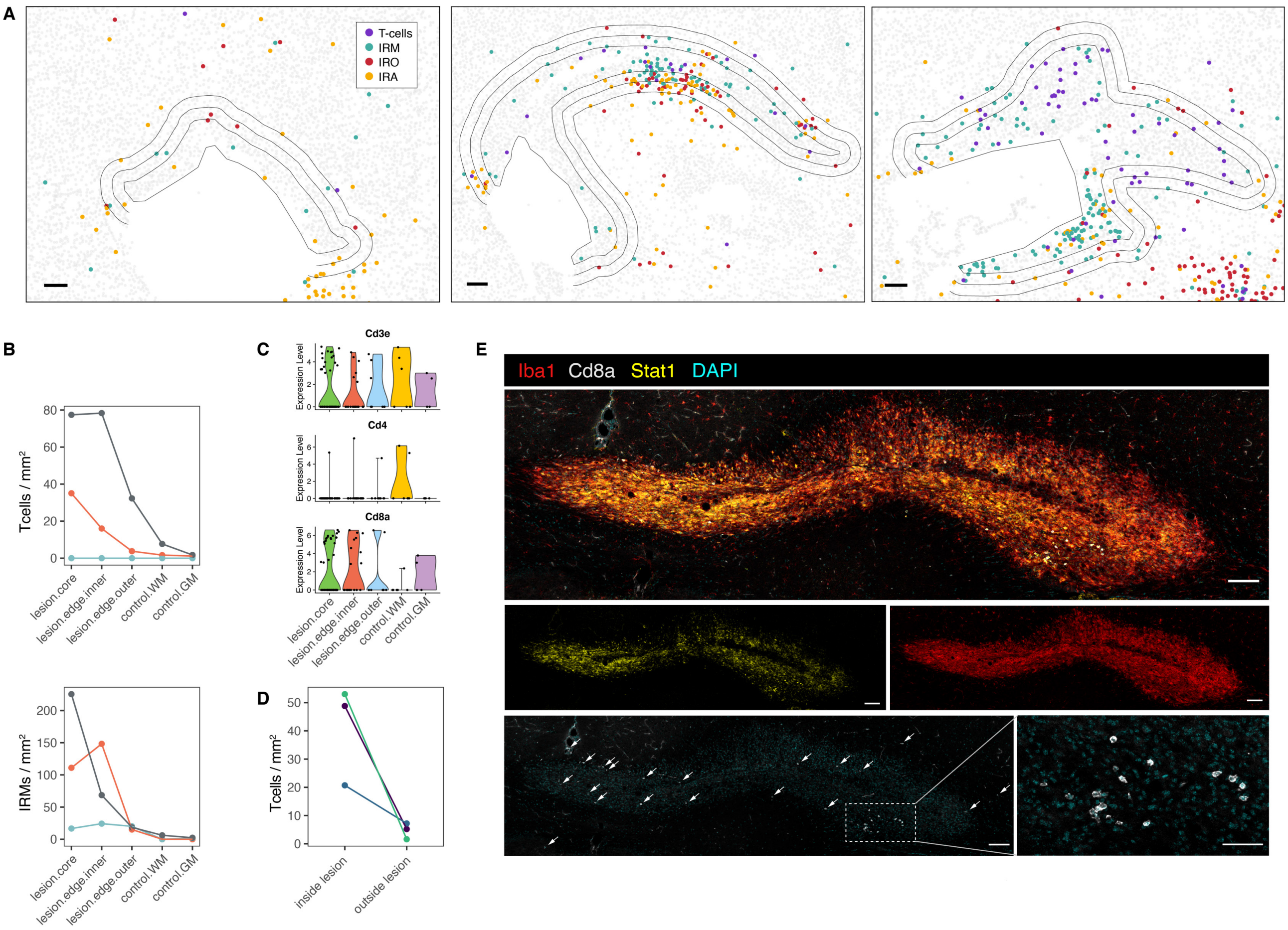

Figure S7. Extended analysis of T-cells and interferon-responsive cell states. A MERFISH spatial plots of T-cells and interferon-responsive glia in the lesion area of three biological replicate sections (top). Polygons depict segmented lesion areas. Scale bar 100 µm. IRM = interferon-responsive microglia, IRO = interferon-responsive oligodendrocytes, IRA = interferon-responsive astrocytes. **B** Density of T-cells (top) and interferon-responsive microglia (bottom) per tissue region in three MERFISH sections. **C** Expression of CD4/CD8 lineage markers in T-cells. **D** Density of CD8-positive T-cells based on immunofluorescence staining. **E** Immunofluorescence staining of IBA1, CD8, and STAT1 in the lesion. Scale bar 100 µm. Representative result of experiment independently repeated in three different animals is shown. Source data are provided as a Source Data file.

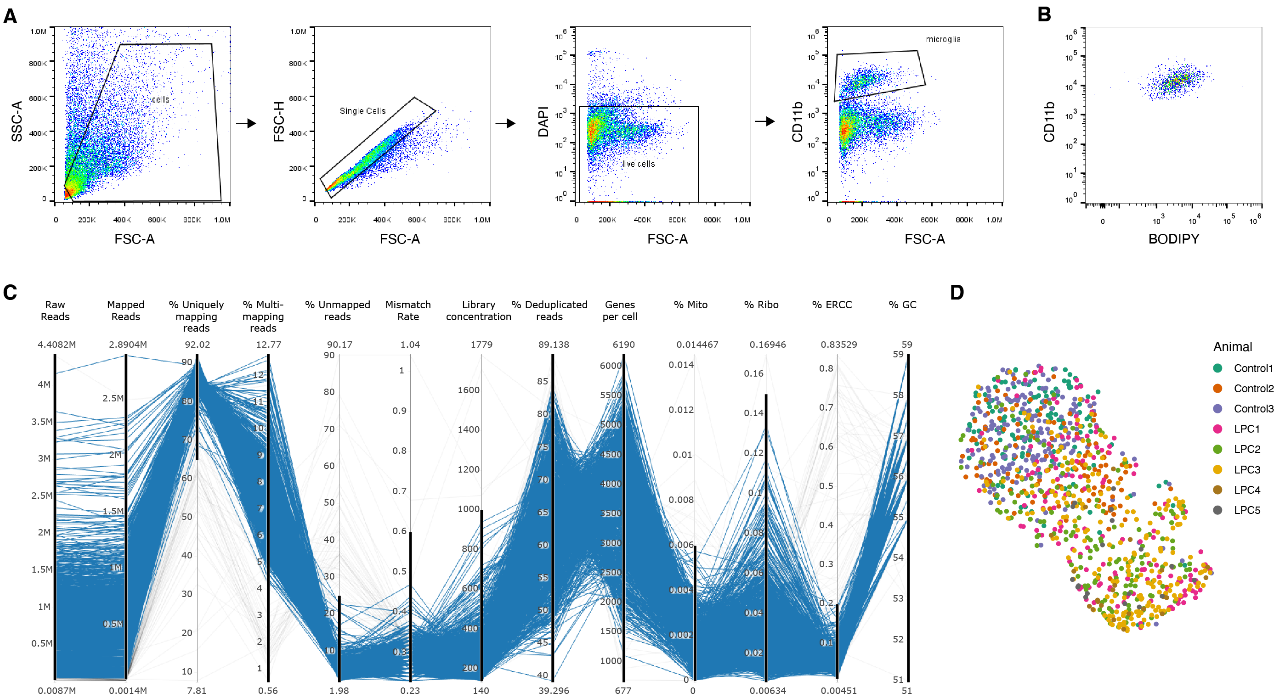

Figure S8. Sorting strategy and quality control of SmartSeq2 data. A Sorting strategy for SmartSeq2 scRNA-Seq. Flow cytometry gating of CD11b positive cells to enrich microglia. **B** Flow cytometry plot of BODIPY fluorescence in cells from LPC-injected animals. **C** Quality control of the SmartSeq2 dataset. Parallel coordinates plot showing cells meeting (blue lines) or failing (grey lines) individual quantitative QC metrics. Bold line segments represent selected threshold boundaries of each QC metric. From left to right, the metrics are: number of raw reads, number of mapped reads, % of uniquely mapping reads, % of multimapping reads, % of unmapped reads, mismatch rate, DNA concentration of final single-cell library, % of deduplicated reads, number of detected genes per cell, % of mitochondrial genes, % of ribosomal genes, % of ERCC spike-ins, % of GC content of reads. **D** UMAP plot of microglial SmartSeq2 data colored by animal.

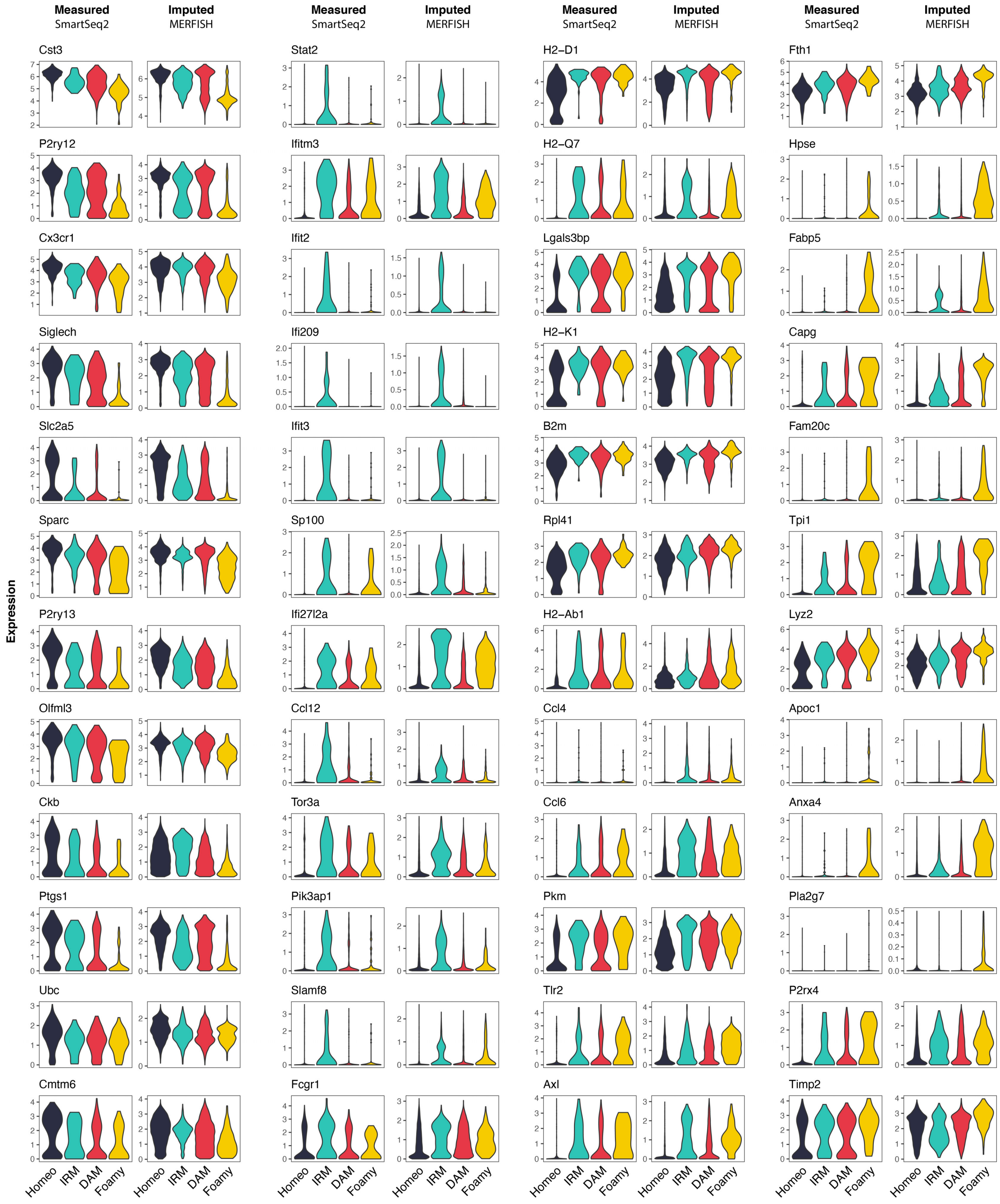

Figure S9. Imputed expression of MERFISH-unmeasured genes. Violin plots showing measured gene expression in SmartSeq2 scRNA-Seq data and corresponding imputed gene expression in MERFISH data across microglial clusters.
