# Supplementary material for: Spatial Transcriptomics-correlated Electron Microscopy maps transcriptional and ultrastructural responses to brain injury
Source: Nat Commun. 2023 Jul 11;14:4115. doi: 10.1038/s41467-023-39447-9 (PMC10336148; doi:10.1038/s41467-023-39447-9)
Supplement: Supplementary file 3 — Description of Additional Supplementary Files [file 41467_2023_39447_MOESM3_ESM.pdf]

Description of Additional Supplementary Files

File Name: Supplementary Data 1
Description: Tables with animal info for MERFISH and SmartSeq2 datasets

File Name: Supplementary Data 2
Description: List of genes targeted in MERFISH experiments

File Name: Supplementary Data 3
Description: Gene expression signatures of identified microglial populations in SmartSeq2 dataset. Related to Figure S3.

File Name: Supplementary Data 4
Description: Gene expression signatures of microglia/macrophages collected from literature. Related to Figure S3E

File Name: Supplementary Data 5
Description: ENRICHR Results of enrichment analysis of lipid-associated microglia signature. Related to Figure S3D.

File Name: Supplementary Data 6
Description: Gene Set Enrichment Analysis results of genes correlating with BODIPY
