# Supplementary material for: Spatial Transcriptomics-correlated Electron Microscopy maps transcriptional and ultrastructural responses to brain injury
Source: Nat Commun. 2023 Jul 11;14:4115. doi: 10.1038/s41467-023-39447-9 (PMC10336148; doi:10.1038/s41467-023-39447-9)
Supplement: Supplementary file 5 — Reporting Summary [file 41467_2023_39447_MOESM5_ESM.pdf]

nature portfolio

Corresponding author(s): Ozgun Gokce

Last updated by author(s): May 25, 2023

Reporting Summary

Nature Portfolio wishes to improve the reproducibility of the work that we publish. This form provides structure for consistency and transparency in reporting. For further information on Nature Portfolio policies, see our Editorial Policies and the Editorial Policy Checklist.

Statistics

For all statistical analyses, confirm that the following items are present in the figure legend, table legend, main text, or Methods section.

n/a	Confirmed	
☐	☒	The exact sample size (*n*) for each experimental group/condition, given as a discrete number and unit of measurement
☐	☒	A statement on whether measurements were taken from distinct samples or whether the same sample was measured repeatedly
☐	☒	The statistical test(s) used AND whether they are one- or two-sided<br>*Only common tests should be described solely by name; describe more complex techniques in the Methods section.*
☒	☐	A description of all covariates tested
☐	☒	A description of any assumptions or corrections, such as tests of normality and adjustment for multiple comparisons
☐	☒	A full description of the statistical parameters including central tendency (e.g. means) or other basic estimates (e.g. regression coefficient) AND variation (e.g. standard deviation) or associated estimates of uncertainty (e.g. confidence intervals)
☐	☒	For null hypothesis testing, the test statistic (e.g. F, t, r) with confidence intervals, effect sizes, degrees of freedom and P value noted<br>*Give P values as exact values whenever suitable.*
☒	☐	For Bayesian analysis, information on the choice of priors and Markov chain Monte Carlo settings
☒	☐	For hierarchical and complex designs, identification of the appropriate level for tests and full reporting of outcomes
☐	☒	Estimates of effect sizes (e.g. Cohen's *d*, Pearson's *r*), indicating how they were calculated

Our web collection on statistics for biologists contains articles on many of the points above.

Software and code

Policy information about availability of computer code

Data collection	Sony SH800S v2.1.6 flow cytometry sofware was used for cell sorting
Data analysis	MERFISH and scRNA-Seq analysis:<br>Seurat package (v 4.1.0) , Monocle3 (v 1.3.1), spatstat R package (v 3.0), BigWarp ImageJ plugin, Morpho R package (v 2.11), spatstat R package (v 3.0), enrichR package (v 3.1), emmeans R package (v 1.8.5), hdWGCNA R package (v 0.2.03), fgsea R package v 1.22), msigdbr R package (v 7.5.1)<br><br>Analysis of IHC data<br>Quantification was performed with ImageJ v 1.53. Bar graphs and boxplots related to IHC staining were generated in R 4.2.3.<br><br>Analysis of EM data<br>The large image stacks were exported as tiles, stitched and aligned using Fiji TrakEM2 1.3.7. Annotation of cell types according to the ultrastructural morphology was performed in VAST v 1.4.1. The VAST object files were exported and reassembled in Blender.

For manuscripts utilizing custom algorithms or software that are central to the research but not yet described in published literature, software must be made available to editors and reviewers. We strongly encourage code deposition in a community repository (e.g. GitHub). See the Nature Portfolio guidelines for submitting code & software for further information.

Data

Policy information about [availability of data](#)

All manuscripts must include a [data availability statement](#). This statement should provide the following information, where applicable:

- Accession codes, unique identifiers, or web links for publicly available datasets
- A description of any restrictions on data availability
- For clinical datasets or third party data, please ensure that the statement adheres to our [policy](#)

The MERFISH and scRNA-Seq datasets generated in this study have been deposited in the Gene Expression Omnibus (GEO) database under accession number GSE202638[https://www.ncbi.nlm.nih.gov/geo/query/acc.cgi?acc=GSE202638]. Figure source data are provided with this paper. All other data that support the findings are available upon request from the authors.

Human research participants

Policy information about [studies involving human research participants and Sex and Gender in Research.](#)

Reporting on sex and gender	not applicable
Population characteristics	not applicable
Recruitment	not applicable
Ethics oversight	not applicable

Note that full information on the approval of the study protocol must also be provided in the manuscript.

Field-specific reporting

Please select the one below that is the best fit for your research. If you are not sure, read the appropriate sections before making your selection.

☒ Life sciences ☐ Behavioural & social sciences ☐ Ecological, evolutionary & environmental sciences

For a reference copy of the document with all sections, see [nature.com/documents/nr-reporting-summary-flat.pdf](#)

Life sciences study design

All studies must disclose on these points even when the disclosure is negative.

Sample size	No power analyses were used to predetermine sample sizes. However, sample sizes were chosen based on prior literature using similar experimental paradigms. (for example doi:10.1016/j.celrep.2019.03.099; doi:10.1016/j.immuni.2017.08.008; doi:10.1016/j.cell.2017.05.018; doi:10.1016/j.neuron.2021.01.027)
Data exclusions	No data were excluded from analysis.
Replication	For all mouse experiments, 2-5 mice per experimental group were analyzed. Replication was successful for all conditions reported.
Randomization	All experimental animals are analyzed. The allocation of samples including brain sections was random.
Blinding	For EM, MERFISH and IHC experiments blinding was not applicable as within-sample comparisons were perfomed. For scRNA-Seq experiments experimentator could not be blinded when preparing samples because of obvious injury site in the brain.

Reporting for specific materials, systems and methods

We require information from authors about some types of materials, experimental systems and methods used in many studies. Here, indicate whether each material, system or method listed is relevant to your study. If you are not sure if a list item applies to your research, read the appropriate section before selecting a response.

Materials & experimental systems

n/a	Involved in the study
☐	☒ Antibodies
☒	☐ Eukaryotic cell lines
☒	☐ Palaeontology and archaeology
☐	☒ Animals and other organisms
☒	☐ Clinical data
☒	☐ Dual use research of concern

Methods

n/a	Involved in the study
☒	☐ ChIP-seq
☐	☒ Flow cytometry
☒	☐ MRI-based neuroimaging

Antibodies

Antibodies used	Plin2 (Novus Biological NB110-40877, 1:200, rabbit), Iba1 (Synaptic Systems 234 009,1:400, chicken), Gpnmb (biotechne, BAF2330, 1:200, goat), Galectin3 (Novus Biological, NBP2-16590, 1:400, rat), Stat1 (Cell Signaling Technology, clone 14994S, rabbit, 1:500) and Cd8a (biolegend, 100702, clone 53-6.7, rat, 1:100)<br><br>secondary antibodies (AlexaFluor™ 488, donkey anti-chicken Invitrogen # A78948, 1:1000, AlexaFluor™555 donkey anti-rabbit, Invitrogen #A-31572, 1:1000, AlexaFluor™ 647 donkey anti-goat, 1:1000 or AlexaFluor™ 647 donkey anti rat, # A48272, 1:1000),<br><br>secondary antibodies against guinea pig (AlexaFluor™ 647, Invitrogen, A-21450, 1:500), rat (AlexaFluor™ 488, Invitrogen, A-11006, 1:500) and rabbit (Goat Anti-Rabbit IgG Antibody (H+L), Biotinylated BA-1000)
Validation	All the antibodies used for IHC were validated by the manufacturers.<br>Primary antibodies:<br>https://www.novusbio.com/products/perilipin-2-adfp-antibody_nb110-40877<br>https://www.sysy.com/product/234009<br>https://www.rndsystems.com/products/mouse-osteoactivin-gpnmb-biotinylated-antibody_baf2330<br>https://www.novusbio.com/products/galectin-3-antibody_nbp2-16590<br>https://www.cellsignal.com/products/primary-antibodies/stat1-d1k9y-rabbit-mab/14994<br>https://www.biolegend.com/fr-ch/products/purified-anti-mouse-cd8a-antibody-157?GroupID=BLG2559<br>Secondary antibodies<br>https://www.thermofisher.com/antibody/product/Donkey-anti-Chicken-IgY-H-L-Highly-Cross-Adsorbed-Secondary-Antibody-Polyclonal/A78948<br>https://www.thermofisher.com/antibody/product/Donkey-anti-Rabbit-IgG-H-L-Highly-Cross-Adsorbed-Secondary-Antibody-Polyclonal/A-31572<br>https://www.thermofisher.com/antibody/product/Donkey-anti-Goat-IgG-H-L-Cross-Adsorbed-Secondary-Antibody-Polyclonal/A-21447<br>https://www.thermofisher.com/antibody/product/Donkey-anti-Rat-IgG-H-L-Highly-Cross-Adsorbed-Secondary-Antibody-Polyclonal/A48272<br>https://www.thermofisher.com/antibody/product/A-11006.html?ef_id=Cj0KCQjwjryjBhD0ARIsAMLvnF_Bfpur_EhOtP8CSnNJvvvY0hFNNPDMkmZVhCWzjq6Bal4ti2zxOeYaAp2YEALw_wcB:G:s&s_kwcid=AL!3652!3!516608152203!!!g!!!12825517856!122158235275&cid=bid_pca_aus_r01_co_cp1359_pjt0000_bid00000_0se_gaw_dy_pur_con&gclid=Cj0KCQjwjryjBhD0ARIsAMLvnF_Bfpur_EhOtP8CSnNJvvvY0hFNNPDMkmZVhCWzjq6Bal4ti2zxOeYaAp2YEALw_wcB<br>https://www.thermofisher.com/antibody/product/Goat-anti-Guinea-Pig-IgG-H-L-Highly-Cross-Adsorbed-Secondary-Antibody-Polyclonal/A-21450<br>https://vectorlabs.com/products/antibodies/biotinylated-goat-anti-rabbit-igg

Animals and other research organisms

Policy information about studies involving animals; ARRIVE guidelines recommended for reporting animal research, and Sex and Gender in Research

Laboratory animals	Male C57BL/6J mice were obtained from Janvier Laboratories. All mice were housed at the animal facility in the German Centre for Neurodegenerative Diseases (DZNE) in Munich in standard, pathogen¬-free conditions. The temperature in the housing unit was kept between 20 and 22 °C with 40–60% humidity and a 12¬-hour light/12-¬hour dark cycle. Animals were 3-4 months old (MERFISH, EM, IHC experiments) or 18 and 24 weeks old (scRNA-Seq).
Wild animals	no wild animals were used in the study
Reporting on sex	All experiments were performed on male animals.
Field-collected samples	no field collected samples were used in the study
Ethics oversight	All mouse experiments in this study were performed with the approval and according to the regulations of the District Government of Upper Bavaria and reported according to guidelines.

Note that full information on the approval of the study protocol must also be provided in the manuscript.

Flow Cytometry

Plots

Confirm that:

☒ The axis labels state the marker and fluorochrome used (e.g. CD4-FITC).

☒ The axis scales are clearly visible. Include numbers along axes only for bottom left plot of group (a 'group' is an analysis of identical markers).

☒ All plots are contour plots with outliers or pseudocolor plots.

☒ A numerical value for number of cells or percentage (with statistics) is provided.

Methodology

Sample preparation	The mice were deeply anesthetized and perfused with cold HBSS between 9am-11am (to decrease circadian fluctuations). Each brain was removed and under a dissection microscope individually micro-dissected; gray matter was isolated from the frontal cortex and white matter form optic tract, medial lemniscus and corpus callosum (attached gray matter and choroid plexus were carefully removed). We used a microglia isolation protocol we previously described26, that prevents ex-vivo transcription and automatizes the mechanical isolation parts using GentleMacs with the Neural Tissue Dissociation Kit (Papain) (Miltenyi Biotec). We added actinomycin D (Act-D, Sigma, No. A1410) to a final concentration of 45 µM into the dissociation solution and enzyme mix to prevent ex-vivo transcription. The dissociated cell suspension was passed through a 70 µm cell strainer (Corning, 352350) before labeling. Subsequently, cells were blocked with mouse FcR-blocking reagent (CD16/CD32 Monoclonal Antibody, eBioscience cat:14-0161-82,1100) and then stained with the antibody against CD11b (PE/Cy7,M1/70, eBioscience, Cat:48-0451-82,1:200) and washed with PBS (Sigma, D8537). Then the cells were then stained with DAPI (4',6-diamidino-2-phenylindole, 1:10000 dilution; Sigma) to label dead cells. Viable (DAPI negative) single immune cells (CD11b positive cells) were sorted by flow cytometry (SH800; Sony).
Instrument	Sony SH800S Cell Sorter
Software	The SH800S software for the data collection. FlowJo was used for the flow cytometry data analysis.
Cell population abundance	During single-cell library preparation and during single cell RNA-Seq analysis sorted cell populations were asessed for quality by measuring DNA concentration and asessing range of transcriptional metrics (details in Methods).
Gating strategy	CNS cells were gated for singlets by using FSC-A and FSC-H, followed by gating for living cell (DAPI negative population), then CD11b-positive cells were sorted.

☒ Tick this box to confirm that a figure exemplifying the gating strategy is provided in the Supplementary Information.
